# Role of Gut Microbiota in the Development and Management of Rheumatoid Arthritis: A Narrative Review

**DOI:** 10.7759/cureus.49458

**Published:** 2023-11-26

**Authors:** Yuti Godha, Sunil Kumar, Anil Wanjari

**Affiliations:** 1 Medicine, Jawaharlal Nehru Medical College, Datta Meghe Institute of Higher Education and Research, Wardha, IND

**Keywords:** treatment, intestinal dysbiosis, development, microbiota, rheumatoid arthritis

## Abstract

Rheumatoid arthritis is an autoimmune condition that damages and inflames the joints. It causes severe disability and lowers the quality of life. While the precise cause of rheumatoid arthritis is still unknown, mounting evidence suggests that the gut microbiota, a diverse colony of bacteria that inhabits the gastrointestinal tract, may play a vital role in the progression and management of this debilitating condition. By evaluating relationships, probable processes, and therapeutic ramifications, this narrative review intends to examine the complex relationship between intestinal microbiota and rheumatoid arthritis. Additionally, for the management of rheumatoid arthritis, the review will assess prospective therapeutic approaches that target the gut flora. Multiple studies have shown that people with rheumatoid arthritis have dysbiosis or an imbalance in their gut microbial ecosystems. Increased intestinal permeability has been linked to changes in the gut microbiota, which allows the transfer of bacterial products into the bloodstream. A search was undertaken through PubMed in June 2023 using keywords like "microbiota", "rheumatoid arthritis" and "treatment". Overall 42 articles were included. Probiotics, prebiotics, and dietary changes are some examples of therapies that can be used to modify the gut microbiota and lessen symptoms, slower the progression of the disease, and enhance therapy results. Understanding the interplay between intestinal microbiota and rheumatoid arthritis will pave the way for innovative and personalized therapeutic interventions that could revolutionize the management of this chronic autoimmune disease.

## Introduction and background

Rheumatoid arthritis is a systemic chronic inflammatory disease that affects approximately 1% of the adult population worldwide [1]. Because the disease targets the self-antigens found in the synovium, cartilage, and bone, it typically results in polyarticular joint damage and functional impairment in the affected patients. The discrepancy in the anti-inflammatory and pro-inflammatory cytokine ratio, brought on by changes in the Th1 cell profile, is what defines the inflammatory pathway in rheumatoid arthritis. In afflicted joint areas, inflammatory cells release tumor necrosis factor alpha (TNF-α), IL-1, and IL-6 while simultaneously releasing IL-11, IL-13, and IL-10 [2]. The incidence of this disease is higher in women than men. Smoking is the environmental component most consistently linked to the development of rheumatoid arthritis, although there are other factors, such as infections and nutrition [2]. The host microbiota, notably the gut microbiota, is critical for the progression of rheumatoid arthritis, even though much research has concentrated on genetic variables [3]. The gut microbiota contributes to the preservation of immunological homeostasis and serves as a gauge of the host's health. This relationship can be disturbed, which can have an impact on mucosal and systemic immunity as well as promote several inflammatory and autoimmune illnesses [4].

The development of more effective treatment options, such as biologics and conventional disease-modifying anti-rheumatic drugs (DMARDs), has significantly improved the prognosis of rheumatoid arthritis in recent decades, despite the disease's high morbidity and mortality rate [4]. This review aims to summarize the current knowledge about gut microbiome, its role, and its application in the pathogenesis of rheumatoid arthritis. In addition, we discuss how gut commensals that function like probiotics influence the microbiome to treat rheumatoid arthritis.

## Review

Methodology

**Figure 1:**
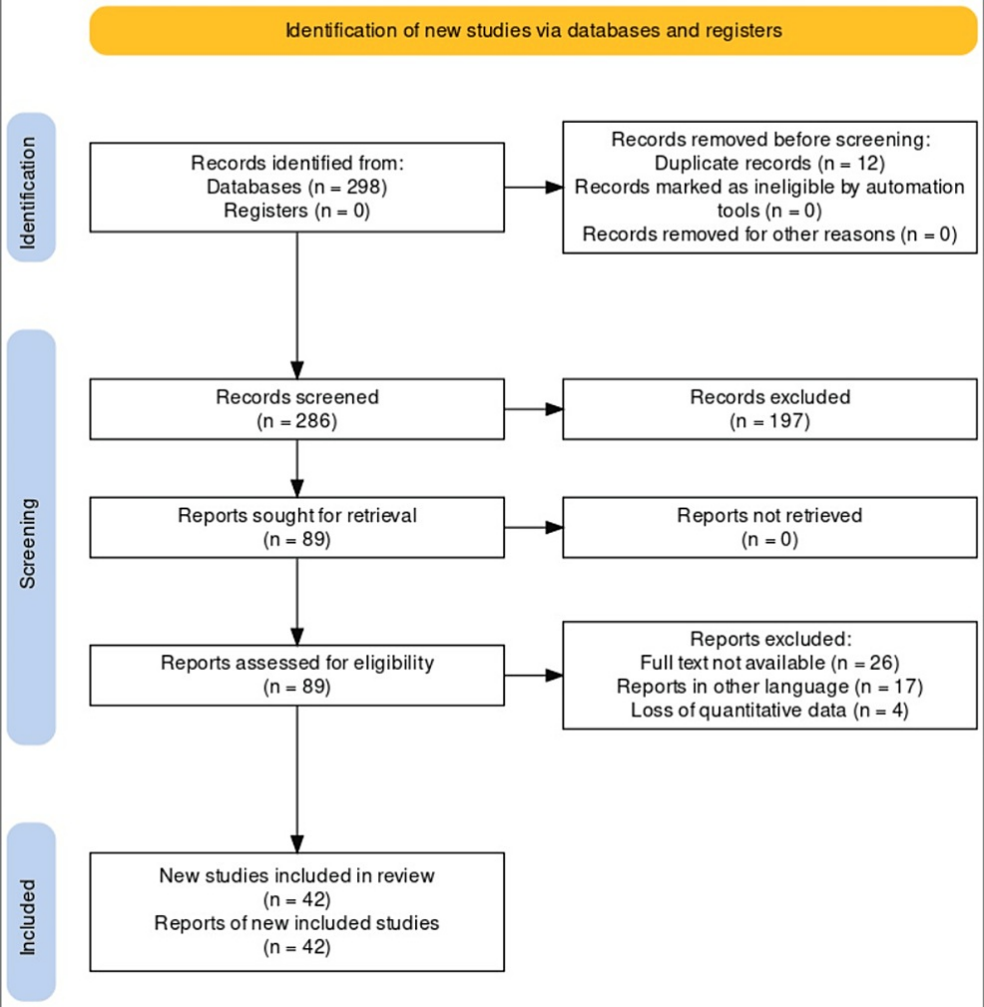
Preferred Reporting Items for Systematic Reviews and Meta-Analyses (PRISMA) flow diagram

The role of gut microbiota in the development and treatment of rheumatoid arthritis was thoroughly reviewed using a literature search. A search was conducted through PubMed in June 2023 using keywords like "microbiota", "rheumatoid arthritis" and "treatment". We also searched bibliographies for important references to pertinent studies. Reference lists of pertinent publications and review papers were manually examined in addition to electronic database searches to find more studies. The selection process for the research that satisfied the inclusion criteria included observational studies, experimental studies, systematic reviews and meta-analyses that looked at the relationship between gut microbiome and its role in developing and treating rheumatoid arthritis. The inclusion of only peer-reviewed and published articles was taken into consideration. Before incorporating the retrieved papers, two reviewers independently examined their titles, abstracts, and complete texts to make sure they complied with the inclusion requirements, and any inconsistencies were settled by discussion and agreement. The steps for study inclusion are depicted in Figure 1.

Development of the gut microbiome

The microbiota begins to form at birth. The general view is that individuals are sterilized before birth. The development of dysbiosis (an imbalance in the gut microbial community, which is associated with a disease) is assumed to be influenced by environmental factors and dietary and nutritional alterations [5]. The infant's intestine is colonized in the early neonatal period when it comes into contact with the mother and the outside world [6]. Infants delivered vaginally have lactobacilli, as well as enterobacteriaceae and bifidobacteria, from breast milk, in their intestines. Skin germs, including Staphylococcus, which are more frequent, are seen in those delivered via C-section [7]. With the help of 22 recently sequenced fecal meta-genomes from people in four different nations and previously released data sets, we were able to find three significant groups that were not specific to any one area or continent [8]. This suggests, even more, the possibility of a small number of stable host-microbial symbiotic states that could react differently to dietary changes and medication usage [9]. The importance of functional analysis to understand microbial communities is highlighted by the fact that diverse species do not always give a wealth of molecular functions [10].

**Figure 2:**
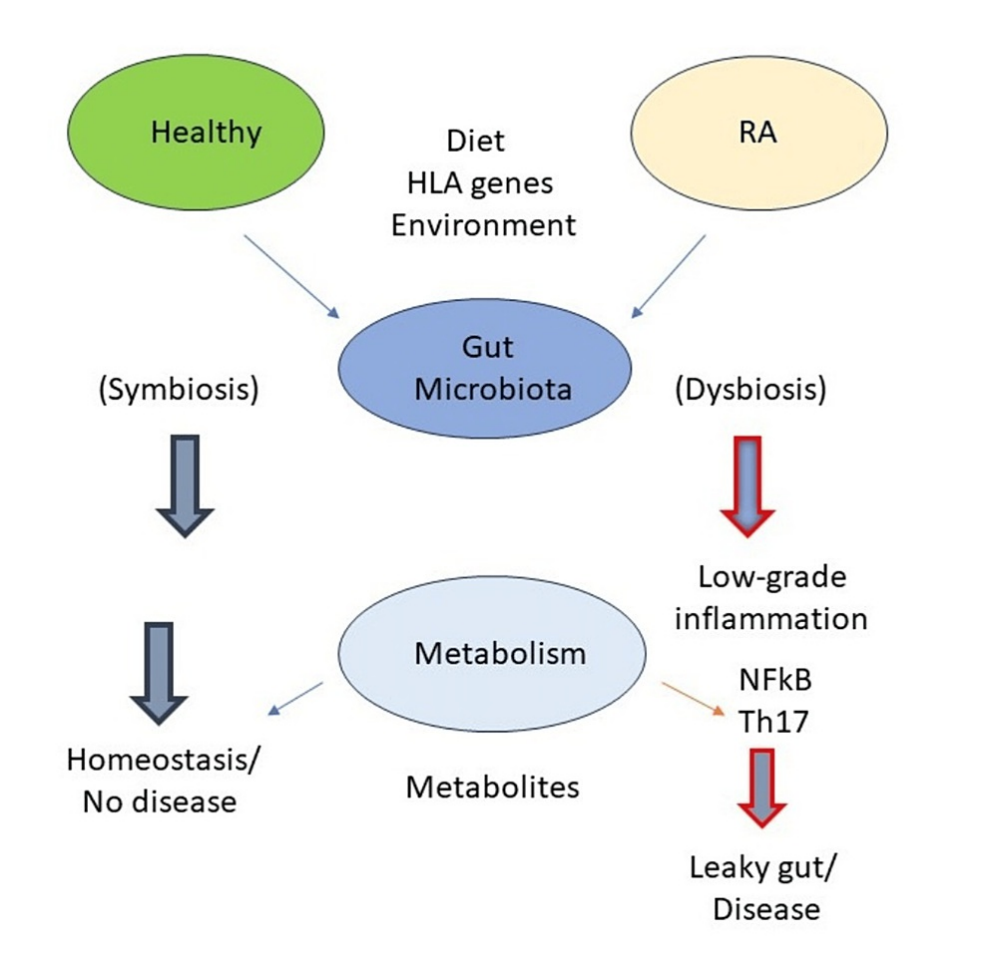
Arthritis susceptibility and the gut microbiome Dysbiosis of bacterial lineages and changes in metabolism of the gut microbiome may drive inflammatory responses that contribute to rheumatoid arthritis [5]. RA, rheumatoid arthritis; HLA, human leukocyte antigen; NFkB, nuclear factor kappa B; TH17, T helper 17 Image credits: Author's own

Environmental variables such as nutrition, illnesses, and smoking can create dysbiosis in the gut microbiota in genetically susceptible individuals, leading to the proliferation or contraction of certain species within a genus. The creation of certain metabolites, nuclear factor kappa B (NFkB) activation, and Th17 cytokine production may all be linked to this dysbiosis. This could compromise the integrity of the gut epithelial layer, leading to a leaky gut and medical disorders as depicted in Figure 2.

Microbiome and host genetic background

The microbial makeup of the gut is significantly influenced by the host's genetic background, particularly by major histocompatibility complex (MHC) genes. The microbiome should now be considered a potential environmental component contributing to disease, complementing earlier genome-wide studies that showed a link between human leukocyte antigen (HLA) genes and vulnerability to autoimmune disease. This higher risk is brought on by aberrant CD4 T-cell activation and the production of autoantibodies [10]. In this instance, a process known as molecular mimicry or cross-reactivity allows infectious pathogens to start the manufacture of antibodies against self-antigens. Another illustration is provided by HLA B27, an HLA with a significant inherited etiology for the pathogenesis of spondyloarthritis, which shares an amino acid sequence with *Klebsiella* nitrogenase and other proteins. Here, antigen mimics cause cross-reactivity in the self-proteins and epitopes of the microbe, causing autoreactive immune responses [11].

Immune Activation in Rheumatoid Arthritis: Mechanisms of Rheumatoid Arthritis Genesis and Maintenance

When the microbiota changes intestinal permeability, zonulin is released, which reduces the function of the intestinal barrier via ejecting the junction complex. The epithelium of the intestine regulates the trafficking of antigens from the lumen to the submucosa in addition to allowing for the absorption of solutes and electrolytes and facilitating food digestion [11]. Both basic scientists and clinicians are now more interested in the new information on the way zonulin works as well as how altered intestinal permeability can contribute to the beginning and development of the disease [12]. It increases the penetration of microbes or their byproducts into the submucosa that can sensitize antigen-presenting cells like dendritic cells or macrophages, in response to these microbes and their byproducts [13]. The connection between the intestinal bacteriome and systemic immune reactions depends on the lymphoid cells. Stimulating inflammatory immune reactions is a different method of rheumatoid arthritis immunopathogenesis [14]. Minor elements of the gut microbiota, including fungi and viruses, as well as the bacteriome, contribute to the activation of the immune system, which may result in the development of rheumatoid arthritis [15].

Treatment

Modulation of the Gut Microbiota as a Potential Rheumatoid Arthritis Therapy

The notion of treatment for rheumatoid arthritis patients has significantly shifted due to the quick development and emergence of disease-modifying medicines [16]. Moreover, these gut microbiome-altering drugs suggest the idea that altering the gut microbiota could be an effective healing or supplemental approach for managing rheumatoid arthritis [17]. There are several ways to influence the microbiome. Omega-3 polyunsaturated fatty acid intake dramatically lowers the levels of inflammation-related indicators, according to numerous meta-analyses [18]. By preventing the intestinal microbiome from destroying the gut mucosa barrier, dietary fiber consumption lowers the risk of pathogen infestation and improves the quality of life [19]. By balancing metabolic dysfunction and managing the intestinal flora, an optimal diet may gradually change the functional state and abnormal immune responses [20]. Certain DMARDs and their effect on microbiota are listed in Table 1.

**Table 1:** List of DMARDs and their effect on microbiota DMARD, disease-modifying anti-rheumatic drug

DMARDs	Study animal	Microbial modulation induced
Methotrexate	Mice; human	After treatment, decrease in *Bacteroides fragilis*; reduced *Enterobacteriaceae* [4].
Sulfasalazine	Human	Decrease in total aerobic bacteria and increase in bacilli [16].
Hydroxychloroquine	Human	Increase in the number and variety of microbes; when combined with doxycycline, it led to a reduced number of bacteria [4].
Azathioprine	Human	Data unavailable
Minocycline	Human	Data unavailable

Role of Lactobacillus rhamnosus

*Lactobacillus rhamnosus* (LR) is a probiotic strain of the *Lactobacillus* genus that has been extensively explored for various human applications. It is a bacteria that has the ability to transfer and metabolize carbohydrates, contributing to the preservation of the integrity of the gut epithelium [21]. Research shows that it reduces bone loss and protects bone health by altering the proportion of Treg-Th17 cells in ovariectomized (Ovx) mice. A recent study showed that LR treatment reduces gut inflammation and enhances intestine barrier function. But LR's immunoregulatory part in governing bone health is still necessary. Sapra et al. examined the immunoregulatory role of LR in Ovx mouse bone health [21]. They showed that osteoclastogenesis is inhibited by LR, which also skews the ratio of Treg-Th17 cells. The equilibrium of the Treg- Th17 cells in the spleen and bone marrow was maintained in mice treated with LR to prevent bone loss and maintain bone mass [22,23].

Role of Hydrolysable Tannins

A hydrolysable tannin (HT), also known as pyrogallol-type tannin, is a kind of tannin that produces gallic or ellagic acids when heated with sulfuric or hydrochloric acids. A variety of vegetable plants, including sumac, tara pods, oak, chestnut, and Aleppo gallnuts, can be used to extract hydrolysable tannins. Gallotannins (GTs) and ellagitannins (ETs) are the two types of HTs, the former are made up of hexahydroxydiphenic acid (HHDP) moieties [24]. GTs release gallic acid (GA) during chemical or enzymatic degradation, whereas ETs also release HHDP, which spontaneously transforms into ellagic acid (EA). GA and EA can be absorbed directly or after being changed by the gut bacteria into pyrogallol derivatives for GA and urolithins for EA [25]. The pathophysiology of rheumatoid arthritis depends on intermediaries from both immunizations [26]. Ellagitannins include punicalagin, which is found in pomegranates. The most studied pure ET for a possible anti-arthritis activity was punicalagin. According to the search, it is clear that hydrotannins are good in reducing inflammation in rheumatoid arthritis, thereby producing an anti-arthritis effect, but a significant problem still exists concerning the necessity for personalized treatment and the security of long-term immunosuppressive therapies [27].

Role of Bovine Lactoferrin

An essential part of the body's defense against infection is known to be played by lactoferrin (LF), which is a globular glycoprotein widely found in various secretions like milk, tears, saliva as well as nasal secretions. Additionally, LF has other beneficial effects, such as anti-inflammatory [28], immunomodulatory, and anticancer effects. LF is a nutrition enhancement diet component and an ingredient used in various cuisines [29]. The small intestine must absorb intact LF without degrading it for it to provide the anti-inflammatory benefits that it does. However, only a limited quantity of orally supplied LF reaches the small intestine intact because the majority is broken down by stomach acid [30]. As a result, different delivery methods have been employed to stop LF breakdown in the stomach. Through the reduction of TNF production, oral treatment of liposomal bovine lactoferrin (LbLF) considerably lessens alveolar bone resorption brought on by stimulation with lipopolysaccharide (LPS) [31]. It was hypothesized that bLF treatment might help stop the pathologic growth of rheumatoid arthritis because TNF is crucial for beginning and advancing inflammation and bone destruction, including periodontitis [32].

Role of Bacteroides fragilis in Enhancing the Healing Effect of Methotrexate

The effects of methotrexate (MTX) on arthritic healing are aided by *Bacteroides fragilis* [33]. DMARDs are frequently utilized as main rheumatic arthritis medications because of their cost-effectiveness and efficacy. Since the 1980s, MTX, one of the DMARDs, has been widely used to treat rheumatoid arthritis [34]. However, MTX's clinical use is still constrained by side effects and unfavorable therapeutic outcomes. With low-dose MTX therapy, more than 75% of patients were found to have several typical adverse effects, including gastrointestinal toxicity, hepatotoxicity, and other issues [35].

Treatment with MTX caused changes in the number of microbes in the gut and a marked decline in the quantity of the well-known human commensal *B. fragilis* [36]. It has been demonstrated that *B. fragilis* inhibits inflammation mediated by T cells and guards against inflammatory conditions of the intestine [37]. An anti-inflammatory response is brought on by the immunomodulatory substance polysaccharide A (PSA) produced by the bacteria [38]. Short-chain fatty acids are another mechanism by which *B. fragilis *controls immune cells in addition to PSA [39]. The bacteria reduced MTX-induced mucositis via modifying macrophage polarization.

Effect of Probiotics on Microbiome

**Figure 3:**
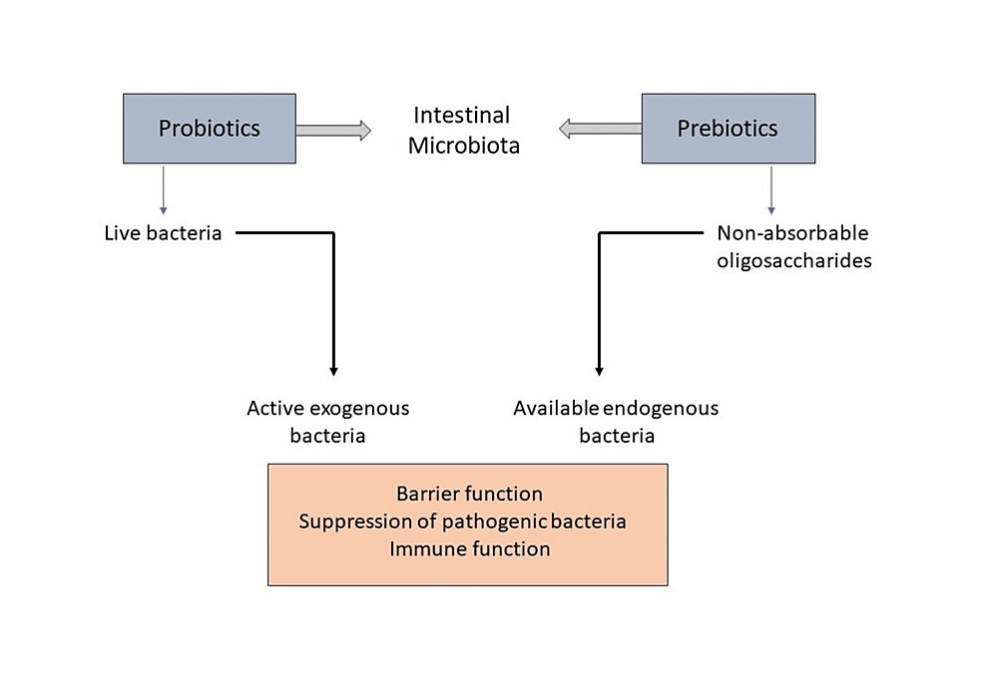
Probiotic and prebiotic concepts: changes to the gut microbiota caused by live bacterial supplements Image credits: Author's own

A variety of living bacteria are referred to as probiotics when they are given to the host in reasonable doses to offer essential health advantages. Probiotics have also been discussed as a budding adjunct for treating rheumatoid arthritis in countless investigations [40]. It is believed that foreign bacteria temporarily alter the gut microbiota, transforming it in ways that can treat and even prevent diseases like rheumatoid arthritis linked to dysbiosis. However, the exact mechanisms underlying these observations are still not fully understood [41]. This impact is related to rheumatoid arthritis patients' cytokine levels being balanced out after treatment. The onset and development of rheumatoid arthritis are influenced by the overexpression of pro-inflammatory cytokines and the downregulation of anti-inflammatory cytokines [42]. Changes to the gut microbiota caused by the effect of probiotics and prebiotics are shown in Figure 3. Table 2 represents a summary of the studies included in the review.

**Table 2:** Summary of the included studies PSA, polysaccharide A; DMARD, disease-modifying antirheumatic drug; SL, sphingolipid; CID, chronic inflammatory disease; SpA, spondyloarthritis; PUFA, polyunsaturated fatty acid; MD, Mediterranean diet; MTX, methotrexate; MAPK, mitogen-activated protein kinase; JAK, Janus kinase

Authors	Year	Findings
Attur et al. [1]	2022	The autoimmune disease rheumatoid arthritis is a long-term condition that damages joints and causes inflammation of the synovium. It is impacted by both genetic and environmental factors.
McInnes and Schett [2]	2011	Rheumatoid arthritis damages polyarticular cartilage and impairs function by attacking self-antigens in joints. Different pro- and anti-inflammatory cytokines, such as TNF-α, IL-1, and IL-6, are produced in injured joints and are indicative of the inflammatory pathway.
Mazmanian et al. [3]	2005	This study highlights the molecular basis of host-bacterial symbiosis and the significance of bacterial PSA produced by *Bacteroides fragilis* in immune system development by identifying a critical role of PSA in forming and regulating the immune system, and showing that a PSA mutant impedes these processes.
Bodkhe et al. [4]	2019	Treatments for rheumatoid arthritis, such as DMARDs like MTX and hydroxychloroquine, affect the host microbiota and immune system, perhaps reversing dysbiosis in rheumatoid arthritis and enhancing disease activity.
Dominguez-Bello et al. [5]	2010	Using 16S rRNA gene pyrosequencing, the research showed that neonates had undifferentiated bacterial populations compared to their mothers, irrespective of the route of delivery. Establishing a baseline for understanding early human microbiome development and its potential impact on child health, vaginally delivered infants mimicked the vaginal microbiota of their mother, while C-section infants resembled skin surface bacteria.
Ardissone et al. [6]	2014	According to the study, meconium from babies born before 33 weeks of pregnancy had higher levels of bacterial 16S rRNA detection; most reads were associated with taxa detected in amniotic fluid. Additionally, specific bacteria exhibited a negative correlation with preterm.
Arumugam et al. [7]	2011	The main factor influencing enterotypes is species composition, which highlights the importance of functional analysis because plentiful molecular functions do not always correspond with numerous species.
Rinninella et al. [8]	2019	For the best immunological and metabolic processes, a varied early-life gut microbiota that is sensitive to environmental cues and is unique maintains a vital host-microorganism balance. The connection between dysbiosis and diseases emphasizes the importance of gut microbiota as a prospective biomarker and a top concern for the inhibition and handling of disease.
Rinninella et al. [9]	2019	A diet rich in animal proteins and saturated fat may encourage the development of harmful microbes in the gut microbiota, whereas an MD supports immune system function, microbial stability, and variety, thereby averting diseases.
Rohrhofer et al. [10]	2021	The metabolism of SLs, which are essential for cell membrane shape and signaling, is impacted by gut flora and nutrition. Diseases are associated with dysregulated SL homeostasis. This highlights the significance of researching microbial SLs and advancing technology to promote immuno-nutrition.
Sturgeon and Fasano [11]	2016	Zonulin is linked to CIDs. Increased levels of zonulin, potentially brought on by gliadin, impair the function of the gut barrier and cause inflammation. Zonulin antagonists like larazotide acetate offer therapeutic potential in a variety of CIDs.
Zaiss et al. [12]	2021	The mucosal immune system and an aberrant local microbiota are thought to combine to cause the chronic autoimmune disease known as rheumatoid arthritis, which mostly affects the joints. The development of rheumatoid arthritis may be influenced by changes in the microbial flora of the mouth, gut, and lungs in both preclinical and clinical cases.
Mauro et al. [13]	2019	The increase in ILC3 that produces IL-17 and IL-22 in the subclinically inflammatory gut of SpA patients is supported by numerous observations. These innate immune cells, which are also seen in healthy entheses, appear to have the capacity to recirculate from the stomach to the inflammatory tissues of SpA patients.
Pianta et al. [14]	2017	*Prevotella copri* appears to have a distinct immunological response to different subgroups of rheumatoid arthritis patients in terms of IgG or IgA. These findings demonstrate the immunological relevance of *P. copri* in the pathophysiology of rheumatoid arthritis.
Wu et al. [15]	2010	Commensal bacteria play a major role in autoimmune diseases. This was shown in a germ-free K/BxN mouse model with decreased arthritis, where the introduction of segmented filamentous bacteria again resulted in the restoration of Th17 cells.
Horta-Baas et al. [16]	2021	The MD may act as an adjunct remedy strategy to improve results in rheumatoid arthritis. The review delves into the possible impacts of components of the MD, including fiber, polyphenols, and n-3 PUFAs.
Dourado et al. [17]	2020	By modifying intestinal microbiota and barrier function, the MD may operate as an adjuvant therapy for rheumatoid arthritis. Components of MD, such as fiber, polyphenols, and n-3 PUFAs, are investigated for their possible role in improving results.
Gioxari et al. [18]	2018	Results from earlier meta-analyses are consistent with the beneficial effects of ω-3 PUFAs on the activity of the rheumatoid arthritis disease. Among the five proinflammatory markers, only leukotriene B4 exhibited a decrease.
Desai et al. [19]	2016	According to the study, in the absence of dietary fiber, the gut microbiota eats host-secreted mucus, weakening the colonic wall and making people more vulnerable to fatal colitis caused by* Citrobacter rodentium*. This provides prospective therapeutic methods through dietary interventions and illustrates the complex relationships between gut microbiome, nutrition, and intestinal health.
Häger et al. [20]	2019	The study explores the relationship between rheumatoid arthritis, gut microbiota imbalance, and low dietary fiber. It suggests that dietary fiber supplementation may enhance regulatory immunological components, which may slow the progression of the disease and improve quality of life.
Sapra et al. [21]	2021	According to the study, *Lactobacillus rhamnosus* may be a promising osteoprotective agent. By regulating osteoclast activity and the balance of Treg-Th17 cells, it can prevent bone loss and preserve density, potentially providing an alternative to expensive and potentially harmful osteoporosis treatments.
Westerik et al. [22]	2018	Human gastrointestinal and urogenital tracts are home to the fermentation-using bacteria *Lactobacillus rhamnosus*. It produces inexpensive, nutrient-dense vegetables in East Africa when mixed with Streptococcus *thermophilus *C106, fostering sustainable development and social benefits—a noteworthy use of natural bacteria for well-being in locations with limited resources.
Mao et al. [23]	2020	Using an inflammation model in piglets, the study showed that *Lactobacillus rhamnosus* GG functions as a probiotic by inhibiting the MAPK and NF-ĸB pathways, suppressing TNF-α, maintaining intestinal integrity, and changing metabolite profiles, such as higher concentrations of ethanolamine and caprylic acid, suggesting potential benefits for intestinal health.
Barnes et al. [24]	2016	Seven gallic acid metabolites were found in the urine, two of which rose following a 10-day mango diet, according to the study. In intestinal-like environments, mango gallotannins release free gallic acid, indicating a potential function as an absorption or microbial metabolism source.
Kawabata et al. [25]	2019	Hydrolysable tannins are converted by polyphenols during intestinal absorption and metabolism to produce a variety of catabolites, such as gallic and ellagic acid. Through their impact on gut flora, proanthocyanidins found in polyphenols show promise in reducing diseases associated with an unhealthy lifestyle.
Giannini et al. [26]	2020	Prospective results in the innate and adaptive immune systems are found in genetic and epigenetic analyses that uncover processes in the genesis of disease. Modifying a disease can be precisely targeted with the help of molecular insights.
Ferro et al. [27]	2017	Effective targeted medicines, such as small compounds like anti-JAK therapies, have been developed in response to recent developments in the pathogenesis of rheumatoid arthritis. Data indicates that these treatments have a good safety record, are highly effective, and have significantly reduced symptoms, stopped radiographic progression, and enhanced quality of life.
Hayashida et al. [28]	2004	Because of its immunomodulatory properties, such as TNF-α suppression and IL-10 upregulation, cow's milk-derived lactoferrin offers a safe natural therapy option. It also shows both preventive and curative effects on adjuvant-induced rheumatoid arthritis in rats.
Lauterbach et al. [29]	2016	Several preventative and therapeutic uses for bovine lactoferrin are demonstrated by its multifunctional activities. Its favorable effect in lowering the prevalence of necrotizing enterocolitis in newborns weighing less than 1250 grams at birth is suggested by preliminary clinical observations.
Troost et al. [30]	2001	According to the study, the adult gastrointestinal tract quickly empties and degrades lactoferrin identified in several test drinks. A considerable amount of lactoferrin taken orally endures stomach breakdown despite being taken orally, indicating possible biological reactions in the gastrointestinal tract.
Bauer et al. [31]	2004	According to the study, gene therapy can successfully treat tachycardia-induced cardiomyopathy in patients with severe atrial fibrillation and left ventricular dysfunction, suggesting that it may be utilized to manage prevalent cardiac arrhythmias in future generations.
Hirota et al. [32]	2007	By stimulating IL-6-driven arthritogenic Th17 cells, genetically modified T cells in mice cause rheumatoid arthritis-like arthritis. Arthritis is inhibited by low levels of IL-17 or IL-6, and worsened by low levels of interferon-gamma. The results recommend therapies for autoimmune disorders, particularly rheumatoid arthritis.
Smolen et al. [33]	2016	A customized treatment plan and prompt diagnosis are essential for managing rheumatoid arthritis, a chronic inflammatory joint disease. Although many patients have positive outcomes, new medicines are still needed. This study covered genetics, pathophysiology, epidemiology, assessment, and treatment techniques.
Bedoui et al. [34]	2019	The mainstay of care for rheumatic conditions, particularly rheumatoid arthritis, is MTX. The review discussed safety in treating virally related rheumatic diseases and examined MTX's function in virally mediated arthritis, anti-inflammatory mechanisms, and potential dangers to immune surveillance.
Wang et al. [35]	2018	For rheumatoid arthritis, MTX is a powerful medication that may also have adverse effects. To maximize effectiveness and minimize side effects, careful administration and additional studies on combination medicines are essential.
Zhou et al. [36]	2022	The toxicity and efficacy of MTX for rheumatoid arthritis are limited. This study suggested new ways to improve the efficacy of MTX in treating rheumatoid arthritis by linking decreasing *B. fragilis* following treatment to side effects and lower efficacy.
Mazmanian et al. [37]	2008	The study showed that *B. fragilis* protects against experimental colitis caused by *Helicobacter hepaticus* by means of its microbial metabolite PSA. Because PSA inhibits pro-inflammatory reactions, it may have a therapeutic value in treating inflammatory diseases in humans.
Su et al. [38]	2020	The study supports employing *B. fragilis* in microecological therapy to alleviate immunological dysfunction and suggests combining microecology with medication to prevent disease recurrence. Additionally, three gut bacteria are identified as potential markers for Graves’ disease.
Zhou et al. [39]	2018	Reviewing the effects of MTX on the gastrointestinal tract and how it affects the gut microbiota, particularly* Bacteroides*, the study suggested supplementing with *B. fragilis* to lessen the damage. Genetic variations may impact the toxicity of MTX, and this study indicated that MTX may indirectly contribute to inflammation in addition to immunosuppression.
Opoku et al. [40]	2022	The study highlighted the possibility of probiotics as adjuvant therapy for symptom reduction while discussing alterations in the gut microbiota associated with rheumatoid arthritis.
Wang et al. [41]	2016	Probiotics may have anti-inflammatory benefits as well as enhance standard of living in people with rheumatoid arthritis, which suggests that they may be useful in mitigating the symptoms of rheumatoid arthritis. Probiotic supplementation is highlighted in the review as a possible adjuvant therapy for rheumatoid arthritis.
Mohammed et al. [42]	2017	Probiotics were used as an adjuvant therapy for rheumatoid arthritis in nine studies comprising 361 patients. The results showed a substantial reduction in IL-6, but an unclear clinical outcome. For clarity, additional high-quality trials are required.

## Conclusions

Rheumatoid arthritis is significantly influenced by the gut microbiome. This review focused on how gut bacteria and medical care interact. Numerous investigations have indicated that individuals with rheumatoid arthritis have an imbalance of the gut microbiota. Rheumatoid arthritis is frequently treated with DMARDs. These medications possess immunomodulatory qualities, but they can also alter the microbiota of the host. Remarkably, the majority of research showed that after taking methotrexate, people's dysbiotic microbiomes were either changed to enhance the quantity of helpful microbial members or partially returned to normal, which was connected to disease activity. Moreover, research on the host-microbiome link in rheumatoid arthritis is required to clarify potential triggers and fill in the information gaps surrounding the function of gene-environment interactions. It will be necessary to do clinical trials on the probiotic strains having this helpful function to make sure they are not harmful. The drug-microbiome network may offer an efficient approach to rheumatoid arthritis therapies in the future, since partial microbial restoration following treatment is linked to treatment efficacy.
